# An Improved Bayesian Shrinkage Regression Algorithm for Genomic Selection

**DOI:** 10.3390/genes13122193

**Published:** 2022-11-23

**Authors:** Jin Zhang, Ling Li, Mingming Lv, Yidi Wang, Wenzhe Qiu, Yuan An, Ye Zhang, Yuxuan Wan, Yu Xu, Juncong Chen

**Affiliations:** 1 College of Science, Nanjing Agricultural University, Nanjing 210095, China; 2 School of Public Health (Shenzhen), Sun Yat-sen University, Shenzhen 518107, China; 3 School of Business Administration, Jiangxi University of Finance and Economics, Nanchang 330013, China; 4 Freshwater Fisheries Research Institute of Jiangsu Province, Nanjing 210017, China; 5 College of Finance, Nanjing Agricultural University, Nanjing 210095, China

**Keywords:** genomic selection, polygenic background, Bayesian, mixed linear model, GEBV

## Abstract

Currently a hot topic, genomic selection (GS) has consistently provided powerful support for breeding studies and achieved more comprehensive and reliable selection in animal and plant breeding. GS estimates the effects of all single nucleotide polymorphisms (SNPs) and thereby predicts the genomic estimation of breeding value (GEBV), accelerating breeding progress and overcoming the limitations of conventional breeding. The successful application of GS primarily depends on the accuracy of the GEBV. Adopting appropriate advanced algorithms to improve the accuracy of the GEBV is time-saving and efficient for breeders, and the available algorithms can be further improved in the big data era. In this study, we develop a new algorithm under the Bayesian Shrinkage Regression (BSR, which is called BayesA) framework, an improved expectation-maximization algorithm for BayesA (emBAI). The emBAI algorithm first corrects the polygenic and environmental noise and then calculates the GEBV by emBayesA. We conduct two simulation experiments and a real dataset analysis for flowering time-related *Arabidopsis* phenotypes to validate the new algorithm. Compared to established methods, emBAI is more powerful in terms of prediction accuracy, mean square error (MSE), mean absolute error (MAE), the area under the receiver operating characteristic curve (AUC) and correlation of prediction in simulation studies. In addition, emBAI performs well under the increasing genetic background. The analysis of the Arabidopsis real dataset further illustrates the benefits of emBAI for genomic prediction according to prediction accuracy, MSE, MAE and correlation of prediction. Furthermore, the new method shows the advantages of significant loci detection and effect coefficient estimation, which are confirmed by The Arabidopsis Information Resource (TAIR) gene bank. In conclusion, the emBAI algorithm provides powerful support for GS in high-dimensional genomic datasets.

## 1. Introduction

Genomic selection (GS) is a method for predicting the genetic value of a test population based on the genomic estimated breeding value (GEBV) predicted from high-density molecular markers positioned throughout the genome [1,2]. Unlike marker-assisted selection, which seeks to identify individual loci significantly associated with the target trait, the GEBV is based on all markers, including both minor and major effect markers [1]. Thus, the GEBV captures more of the genetic variation, even minor variation for the target trait under selection, potentially leading to more rapid and lower-cost gains from breeding. In the past decades, GS has been widely used in the genetic dissection of animals and plants [1,2,3,4], and it also plays a curial role in genetics and breeding for important quantitative traits of animals and plants.

Powerful prediction ability is a prerequisite for the successful application of GS, which is influenced by several factors, including sample size, the genetic structure of traits, marker density, statistical algorithm, genetic background and so on. The advanced statistical algorithm is an intuitive and essential way to improve the prediction accuracy for breeders. In recent decades, a number of statistical methods have been proposed for GS, including best linear unbiased prediction (BLUP), genomic BLUP (GBLUP [5]), least absolute shrinkage selection operator (LASSO [6]) and elastic net [7]. The Bayesian alphabet models have been developed at the same time and assume that SNP effects follow different distributions, including BayesA, BayesB [8], BayesC, BayesCπ [9] and BayesR [10]. They achieve increased accuracy of genomic prediction, whereas Markov chain Monte Carlo (MCMC) sampling is time-consuming [11,12,13]. Usually, the implementation of Gibbs sampling for the above methods improves the computing speed; however, it is still slow in the case of large numbers of SNPs genotyped in huge individuals [12]. To reduce the computing time and improve the accuracy of the GEBV for Bayesian methods [11,14], several algorithms have been proposed besides Gibbs sampling. VanRaden [5] developed an iterative approximation algorithm of both BayesA and BayesB. Meuwissen [11] proposed a new fast iterative algorithm called fastBayesB, based on BayesLASSO. Hayashi and Iwata [15] implemented the EM algorithm to BayesA, which improved the efficiency of GEBV estimations. Wang and Chen [12] developed a fast EM counterpart to MCMC BayesR. Zhao [16] proposed a fast parallel Bayesian regression algorithm (BayesXII) for models, which greatly reduces the computation time while guaranteeing the same accuracy as conventional samplers. Breen [17] introduced the blocked Gibbs sampling to greatly reduce the computational time. Though all of the above statistical methodologies have been proposed to greatly reduce computation time, they ignored the genetic background in the linear model for GS.

Since the introduction of the mixed linear model (MLM) approach [18,19,20,21] to the concept of quantitative trait analysis, the dissection ability has been significantly increased. Zhou developed BSLMM [21], which is a hybrid of two widely used models, MLM and Bayesian variable selection regression model. Wang proposed a computationally efficient Bayesian algorithm emBayesR [12], which assumes the combined effect of the other SNPs as a residual breeding value. Xu [22] developed a powerful and efficient Bayesian GS model, FMixFN, by using four zero-mean-normal distributions as prior distributions. For all the above methods, the SNP effect is considered as the fixed effect in the model, which may not match the genetic structure and is disadvantageous to the estimation of GEVB [23,24,25,26].

In this study, the population structure and polygenic background are considered in the mixed model, which can effectively improve the accuracy of GS. We propose a two-stage flexible approach to GS to estimate the GEBV. In our mixed linear model, all SNP effects are considered random effects. In the first stage, the emBAI algorithm first whitens the covariance matrix of the polygenic and environmental noise. Subsequently, the emBayesA method is applied to implement estimation and prediction. In this study, a series of simulated experiments and real datasets analyses are conducted to illustrate the advantages of this new method. For comparison, the established methods [27], including expectation maximization BayesA (emBA), expectation maximization elastic-net (emEN), expectation maximization Bayesian ridge regression (emRR), expectation maximization Gaussian maximum likelihood (emML) and expectation maximization BayesC (emBC) are used for analyzing the above datasets.

## 2. Materials and Methods

### 2.1. Genetic Model

For the following mixed linear model, assuming *n* individuals and *p* genetic markers, *p* >> *n*, it can be described as:

(1) y=Wα+Zγ+u+ε

where $y={\left(y_{1},\ldots ,y_{n}\right)}^{T}$, $y_{i}$ is the phenotype of the *i*th individual in a sample of size *n*; *α* is a c×1 vector of the fixed effects, including the intercept, population structure effect and so on; *W* is the corresponding designed matrix for α; *Z* is an n×1 vector of marker genotypes, and $\gamma \sim N\left(0,\sigma_{\gamma }^{2}\right)$ is a random effect of each marker; $\sigma_{\gamma }^{2}$ is the variance of γ; $u\sim MVN\left(0,\sigma_{g}{}^{2}K\right)$ is an n×1 random vector of polygenic effects, $\sigma_{g}{}^{2}$ is the variance of polygenic background, and *K* is a known n×n relatedness matrix; $\varepsilon \sim MVN\left(0,\sigma^{2}I_{n}\right)$ is an n×1 vector of residual errors; $\sigma^{2}$ is the variance of residual error; $I_{n}$ is a n×n identity matrix. *MVN* denotes multivariate normal distribution.

As γ is treated as being a random effect, the variance of *y* in the model (1) is:

(2) $$\mathrm{var}(y)=\sigma_{\gamma }^{2}ZZ^{T}+\sigma_{g}{}^{2}K+\sigma^{2}I_{n}=\sigma^{2}\left(\lambda_{\gamma }ZZ^{T}+\lambda_{g}K+I_{n}\right)$$

where $\lambda_{\gamma }=\sigma_{\gamma }{}^{2}/\sigma^{2}$, $\lambda_{g}=\sigma_{g}{}^{2}/\sigma^{2}$, which are the two ratios of variance components, respectively.

### 2.2. The Improved EM Algorithm for BayesA (emBayesAI) Algorithm

The emBayesAI algorithm is a two-stage approach for GS, which simultaneously estimates regression effects and calculates the GEBV. We describe the stages as follows:

#### 2.2.1. The Polygenic and Residual Noise Whitening Stage

The estimations of two ratios $\lambda_{\gamma }$ and $\lambda_{g}$ cause an expensive computational burden. The polygenic variance is always larger than zero, whereas we assume $\lambda_{\gamma }=0$, since most markers are not associated with the trait. Therefore, we estimate ${\hat{\lambda }}_{g}$ by the reduced model (1), which removes Zγ with only polygenic background, and replace $\lambda_{g}$ in (2) by ${\hat{\lambda }}_{g}$ [25,28], avoiding time-consuming a re-estimate of $\lambda_{g}$ for each single marker scanning. Thus,

(3) $$\mathrm{var}(y)=\sigma^{2}\left(\lambda_{\gamma }ZZ^{T}+{\hat{\lambda }}_{g}K+I_{n}\right)=\sigma^{2}\left(\lambda_{\gamma }ZZ^{T}+B\right)$$

An eigen (or spectral) decomposition of the positive definite matrix $B={\hat{\lambda }}_{g}K+I_{n}$ is:

(4) $$B=Q\Lambda Q^{T}=\left(Q\Lambda^{\frac{1}{2}}Q^{T}\right)\left(Q\Lambda^{\frac{1}{2}}Q^{T}\right)$$

where Q is orthogonal, Λ is a diagonal matrix with positive eigenvalues. Let $C=Q\Lambda^{-\frac{1}{2}}Q^{T}$, the model (1) is changed to:

(5) $$y_{c}=W_{c}\alpha +Z_{c}\gamma +\varepsilon_{c}$$

where, $y_{c}=Cy$, $W_{c}=CW$, $Z_{c}=CZ$, $\varepsilon_{c}=Cu+C\varepsilon \sim MVN\left(0,\sigma^{2}I_{n}\right)$ [26,28].

#### 2.2.2. EM Algorithm for BayesA Stage

In the emBayesA method [8,15], we focus on the following linear model,

(6) $$y_{c}=W_{c}\alpha +Z_{c}\gamma +\varepsilon_{c}$$

where $y_{c}$ is the phenotypic value after polygenic background correction, which is the same as it in the model (5). *α* is a vector of fixed non-genetic effects, including the population mean, and *W_c_* is the corresponding corrected design matrix. γ is a *q* × 1 random effect vector, *q* is the total number of SNP on the whole genome, $Z_{c}$ is the corresponding corrected genotypic matrix for γ. The prior distribution the SNP effect is $\gamma_{k}\sim N\left(0,\sigma_{k}^{2}\right),k=1,\ldots ,q$, $\sigma_{k}^{2}\sim \chi^{-2}\left(v,S\right)$. The parameter set in the emBayesA method is denote as $\theta =\left(\alpha ,\gamma_{1},\gamma_{2},\ldots ,\gamma_{k},\ldots ,\gamma_{q},\sigma_{1}^{2},\sigma_{2}^{2},\ldots ,\sigma_{k}^{2},\ldots ,\sigma_{q}^{2},\sigma^{2}\right)$. To accelerate the computing speed, Hayashi and Iwata [15] regard the variances of SNP effects $\sigma_{k}^{2}$ as missing data and replace them with the conditional posterior expectation,

(7) $${\hat{\sigma }}_{k}^{2}=\frac{{\hat{\gamma }}_{k}^{2}+S}{v+1}$$

The log-posterior distribution of θ is,

(8) $$\begin{array}{c} \log p\left(\theta |y_{c},Z_{c}\right)\propto -\frac{n}{2}\log\left(\sigma^{2}\right)-\frac{\sum_{i=1}^{n}{\left(y_{c_{i}}-\sum_{j=1}^{c}w_{ij}\alpha_{j}-\sum_{k=1}^{q}z_{c_{ik}}\gamma_{k}\right)}^{2}}{2\sigma^{2}}.\\ -\sum_{k=1}^{q}\left\{\left(\frac{v+3}{2}\right)\log\left({\hat{\sigma }}_{k}^{2}\right)+\frac{\gamma_{k}^{2}+s}{2{\hat{\sigma }}_{k}^{2}}\right\} \end{array}$$

Maximizing the log-posterior and making the partial derivative with respect to each parameter equal to 0, the estimations are shown as the following equations:


(9) $${\hat{\gamma }}_{k}=\frac{\sum_{i=1}^{n}z_{c_{ik}}\left(y_{c_{i}}-\sum_{j=1}^{c}w_{ij}{\hat{\alpha }}_{j}-\sum_{m\neq k}z_{c_{im}}{\hat{\gamma }}_{m}\right)}{\sum_{i=1}^{n}z_{c_{ik}}^{2}+\sigma^{2}/{\hat{\sigma }}_{k}^{2}}$$

(10) $${\hat{\alpha }}_{j}=\frac{\sum_{i=1}^{n}w_{ij}\left(y_{c_{i}}-\sum_{h\neq k}w_{ih}{\hat{\alpha }}_{h}-\sum_{k=1}^{q}z_{c_{ik}}{\hat{\gamma }}_{k}\right)}{\sum_{i=1}^{n}w_{ij}^{2}}$$

(11) $${\hat{\sigma }}^{2}=\frac{\sum_{i=1}^{n}{\left(y_{c_{i}}-\sum_{j=1}^{c}w_{ij}{\hat{\alpha }}_{j}-\sum_{k=1}^{q}z_{c_{ik}}{\hat{\gamma }}_{k}\right)}^{2}}{n}$$

The EM algorithm for BayesA is summarized as follows:

1. E-step: $\sigma_{k}^{2}$ is estimated as ${\hat{\sigma }}_{k}^{2}$ shown in (7).
2. M-step: updated ${\hat{\gamma }}_{k},\;\;{\hat{\alpha }}_{j}\;$and ${\hat{\sigma }}^{2}$ are given according to (9), (10) and (11).

The E-step and M-step are repeated until the convergence criterion is satisfied. In the study, we adopt the maximum iterations equal to 200 for the EM algorithm [27].

### 2.3. Comparison Methods

**Expectation maximization BayesA (emBA** [15]) assumes that all effects of SNP follow a normal distribution, the variances of which are assigned a scale inverse chi-square distribution, and the hyperparameters are directly related to genetic structure. Compared with the MCMC-based BayesA, the prediction accuracy and computational speed of emBA are improved.

**Expectation maximization elastic-net (emEN** [29]) is a linear regression model that uses $L_{1}$ and $L_{2}$ priors as regularization matrices, which solves the elastic net model using a Gibbs sampler. It is more flexible under the condition of predictors with more parameters than the sample size.

**Expectation maximization BayesC (emBC** [9]) assumes that only a small proportion of loci are associated with a trait (have non-zero effects), whereas most others are not; meanwhile, their ratio is unknown. The emBC has an obvious computational advantage over the MCMC-based BayesC, and it becomes significant as the number of markers increases.

**Expectation maximization Gaussian maximum likelihood (emML** [27]) is a classical GS estimation algorithm. The ML focuses on evaluating a set of parameters under the maximum probability. It is a simple, stable and easily operative approach under the Bayesian framework, and it shows excellent estimation properties, as well [30].

**Expectation maximization Bayesian ridge regression (emRR** [31]) assumes that all regression coefficients have equal variance. It introduces the regular term automatically in the estimation process, which finally obtains the posterior distribution of the parameters, avoiding overfitting in the large-scale likelihood estimation.

All of the above methods were implemented by the R software package *bWGR* (http://github.com/cran/bWGR (accessed on 5 June 2021)).

### 2.4. Experimental Materials

#### 2.4.1. The Simulation Data

To illustrate the advantages of the emBAI algorithm, we performed two Monte Carlo simulation experiments and measured all algorithms from the perspectives of prediction accuracy, MSE, MAE, AUC and correlation of prediction. We generated genotypes according to the minor allele frequency (MAF) in the interval [0.1, 0.5] under Hardy–Weinberg equilibrium. The simulated dataset contained 2000 individuals with 10,000 genetic variants generated by MLM. The population mean was set to 10.0, and the residuals were set to 10.0.

We conduct two simulations in this study. For the first simulation, only one fixed position QTN placed on the 98th marker with 0.1 heritability was simulated. For the second simulation experiment, we randomly selected 50 QTNs. The MAF of each QTN is larger than 0.3, and the total phenotypic variance of all QTNs was 0.5. Each simulation experiment was repeated 100 times. For each simulation, we further considered nine scenarios of three background noise by three sample size combinations, including two, five and ten times polygenic background and 500, 1000 and 2000 sample sizes.

#### 2.4.2. The *Arabidopsis* Data

**Figure 1:**
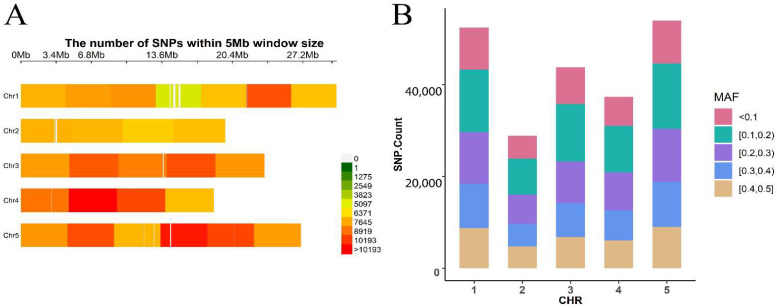
Marker density (**A**) of *Arabidopsis* natural population showing with color lumps with 1 Mb window size. MAF (**B**) of *Arabidopsis* natural population whole genomic SNPs for 5 chromosomes.

The real dataset included 199 *Arabidopsis* inbreed lines (http://www.arabidopsis.usc.edu/ (accessed on 5 January 2022)), which contain 216,130 SNPs and 107 traits [32]. Among these traits, two traits related to flowering time are used to validate the performance of various methods in this study, including (1) LD: days to flowering under long days; and (2) FT22: days to flowering at 22 °C. After quality control and removing the missing data, the datasets left us with 167 individuals and 193 individuals for LD and FT22, respectively, and the density of SNPs and MAF are shown in Figure 1. Both phenotypes and genotypes were obtained from http://www.arabidopsis.usc.edu/ (accessed on 22 January 2022).

#### 2.4.3. Evaluation Indicators

In this study, MSE, MAE, Pearson correlation coefficient ***r*** and AUC were selected to evaluate the performance of all methods.

***MSE*** is defined as:

(12) $$MSE=\frac{1}{n}\overset{n}{\underset{i=1}{\sum }}{\left(y_{i}-{\hat{y}}_{i}\right)}^{2}$$

***MAE*** is defined as:

(13) $$MAE=\frac{1}{n}\overset{n}{\underset{i=1}{\sum }}\left|y_{i}-{\hat{y}}_{i}\right|$$

Both *MSE* and *MAE* are common indexes to evaluate the prediction accuracy in regression model. Smaller values of *MSE* and *MAE* indicate higher accuracy of the results. Assuming that $y_{i}\left(i=1,2,\ldots ,n\right)$ is the phenotypic observation of quantitative traits, ${\hat{y}}_{i}\left(i=1,2,\ldots ,n\right)$ is the predicted value.

The Pearson correlation coefficient ***r***is defined as:

(14) $$r=\overset{n}{\underset{i=1}{\sum }}\left(\frac{y_{i}-\bar{y}}{s_{y}}\right)\left(\frac{{\hat{y}}_{i}-\bar{\hat{y}}}{s_{\hat{y}}}\right)$$

where $\bar{y}=\frac{1}{n}\overset{n}{\underset{i=1}{\sum }}y_{i}$, $\bar{\hat{y}}=\frac{1}{n}\overset{n}{\underset{i=1}{\sum }}{\hat{y}}_{i}$, $s_{y}=\sqrt{\frac{\sum_{i=1}^{n}{\left(y_{i}-\bar{y}\right)}^{2}}{n-1}}$, $s_{\hat{y}}=\sqrt{\frac{\sum_{i=1}^{n}{\left({\hat{y}}_{i}-\bar{\hat{y}}\right)}^{2}}{n-1}}$. The Pearson correlation coefficient ranges from −1 to 1 and reflects the proximity between predicted and true value, so that the closer |r| is to 1, the stronger the linear relationship between two values.

***AUC*** is defined as the area under the receiver operating characteristic curve (ROC), which illustrates the prediction ability of model. The closer AUC is to 1, the more powerful of the predictive algorithm is.

## 3. Results

### 3.1. Simulation Studies

First, we compare the model performance of emBAI to the established approaches, including emBA, emEN, emRR, emML and emBC, through two Monte Carlo simulation experiments. The simulation datasets contained 2000 individuals with 10,000 genetic variants. For the two simulated experiments, we considered one fixed-position QTN and 50 randomly selected QTNs, respectively.

**Figure 2:**
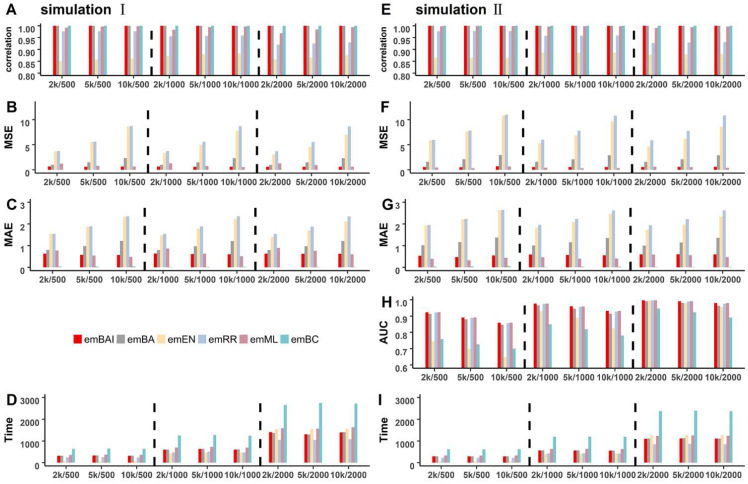
The correlation (**A**,**E**), MSE (**B**,**F**), MAE (**C**,**G**), AUC (**H**) and computing time (**D**,**I**) of two simulation experiments using emBAI, emBA, emEN, emRR, emML and emBC.

For the first simulation experiment, Figure 2A shows the correlation between phenotypes and the GEBV obtained by each method. Figure 2B,C illustrate both the MSE and MAE of prediction. Obviously, emBAI, emBA and emBC have the highest correlation of nearly 1 under various scenarios (Figure 2A), followed by emML and emRR (about 0.97 and 0.95), whereas emEN has a relatively lower correlation, about 0.86. The performance of emBAI is slightly better than that of emBA. Obviously, the correlation of each algorithm increases as the sample size increases, and the background noise has a very mild effect on emBayesAI and other methods. As the results of MSE and MAE show (Figure 2B,C), the prediction for emBC is much more accurate, followed by emBAI, emML and emBA, which are in the same magnitude, while emEN and emRR show a relatively lower accuracy compared with the other algorithms. Meanwhile, the accuracy of all the algorithms decreases as background noise increases, which means that the background noise has a significant impact on the predictive accuracy.

For simulation experiment 2, there are 50 random QTNs associated with the phenotype. The results are shown in the right panel of Figure 2 (Figure 2E–G). The tendency of the correlation and accuracy between phenotypes and the GEBV is similar to that of simulation 1. The results reveal the superiority of emBAI, emBA and emBC in correlation and accuracy under multiple QTNs, followed by emML, emRR and emEN. Furthermore, we apply the AUC of regression coefficients for the different methods. For only one QTN simulated in experiment 1 are all AUC not shown to be nearly one. Figure 2H shows the average AUC of all the methods for 100 replications. The AUC estimates of emBAI are much higher than those obtained by the other methods: its average AUC is 0.95, followed by emML (0.945), emRR (0.943) and emBA (0.93), while emBC (0.82) and emEN (0.85) perform poorly compared with the other methods. Notably, emBAI increases the AUC by 10% more than emBC and emEN in all scenarios. As the Figure illustrates, the sample size and genetic background play an important role in the AUC estimates. The performances of all the methods are significantly improved when the sample size increases and the genetic background decreases. The new method, emBAI, increases the predictive ability from multiple perspectives.

In addition, we compared the computing times for the simulation experiments under 100 replications (Figure 2D,I). Among all methods, emBAI, emBA, emRR and emML have relatively stable, fast computing speeds under each scenario. They require about 1000 s for 2000 individuals (Intel Xeon E5-2630 v4, CPU 2.20 GHz, Memory 64 G). Meanwhile, emEN has a faster computing speed under the small sample size scenarios, showing a dramatic increase in time as the sample size increases, which indicates that emEN is slow with large numbers of individuals. The emBC requires the most expensive computing time at about 1 h for 2000 individuals, which is nearly two times more than the emBAI algorithm.

### 3.2. The Arabidopsis Data Analysis

To obtain further insights into the accuracy of the GEBV among emBAI and the established methods, emBA, emEN, emRR, emML and emBC, we analyze two traits related to flowering time in *Arabidopsis* dataset [32], including LD and FT22.

**Figure 3:**
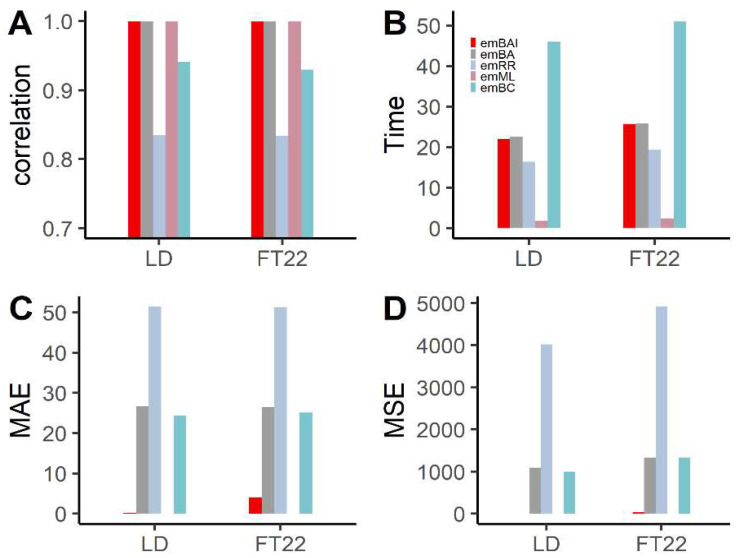
The correlation (**A**), computing time (**B**), MAE (**C**) and MSE (**D**) of *Arabidopsis* natural population analyses using emBAI, emBA, emRR, emML and emBC.

Figure 3 shows the accuracy of the five methods for the two flowering-related traits. The correlation between the phenotypes and the GEBV from emBAI is consistently much higher (Figure 3A), followed by emML and emBA. The correlation of these three methods is over 0.99, significantly higher than those obtained by emRR and emBC. The pattern of accuracy is similar to correlation, as the estimations of emBAI and emML are more accurate (Figure 3C,D) and have less MSE and MAE, followed by emBA, emBC and emRR. The results of real data analysis, correlation and accuracy are slightly different from the simulation experiences. The performance of emBC is more inaccurate than in the simulation, mainly owing to the fact that emBC hardly captures the complicated genetic background. We also analyzed the *Arabidopsis* data using the emEN method, which failed to generate any meaningful results (all estimated effects are NA; data not shown). It may owe to the fact that the emEN method is not flexible enough to analyze the “big P, small N” datasets. Therefore, emBAI is more robust than the other methods from multiple perspectives.

According to the computing speed of the five different methods, emML is computationally much faster than the other methods. The computing times of emBAI, emBA and emRR are on the same order of magnitude, which requires less than 25 s. The emBC method is much more time-consuming than the other four methods and takes over two times more computing time than the emBAI algorithm. The emBAI method has a more stable computing time in simulation experiments and real data analysis. Overall, the emBAI algorithm is recommended in terms of correlation, prediction accuracy and computing speed across all experiments.

To further validate the emBAI algorithm, the top 20 putative QTNs of different methods are used to mine the candidate genes from TAIR (https://www.arabidopsis.org/ (accessed on 13 March 2022)). The quantity of confirmed genes is listed in Table 1. The emBAI algorithm detects 10 confirmed genes, which are associated with the flowering time traits, while the other methods, emBA, emBC, emML and emRR, detect 10, 6, 2 and 4, respectively. Though the quantity detected by emBAI is the same as that detected by emBA, emBAI dissected three clusters of genes that were associated with the same trait (Table 1), including genes AT2G38185, AT2G38195 and AT2G38220, adjacent to the SNP located at 16,020,457 bp on chromosome 2, which were found to relate to long days (LD). More importantly, genes AT2G38185, AT2G38195 and AT2G38220 were simultaneously detected by two traits of LD and FT22. Moreover, these three detected genes associated with target traits could be simultaneously detected by emBC. Obviously, for emBAI, the ability to detect significant genes (confirmed by TAIR) related to the relevant flowering traits is more powerful. It illustrates that emBAI is more accurate, powerful and efficient than the other methods, according to its correlation, MSE, MAE and detection capabilities.

## 4. Discussion

As advanced biological technology generates millions of molecular markers and large-scale SNP markers, traditional computational models show their limitations, proving to be inflexible and time-consuming in the face of big data. Therefore, the development of new computational models to improve accuracy and computational efficiency is an urgent problem in GS. The purpose of this study is to propose a new optimization algorithm in the framework of Bayesian shrinkage regression in order to improve accuracy and computational efficiency in the estimation of the GEBV for important quantitative traits. All the results demonstrated that the new method, emBAI, replaces Gibbs sampling with EM estimation and improves the computational speed compared with the existing methods. Meanwhile, the method takes into account the population structure and polygenetic background, which makes it more accurate and efficient in terms of prediction accuracy, mean square error and correlation of predictions. These provide accurate algorithms and an accurate theoretical basis for GS in high-dimensional genomic datasets.

The accuracy of GEBV estimation plays an important role in GS implementation, and the core of the estimation is the statistical algorithm. In this study, we propose a new algorithm, the improved EM algorithm for BayesA (emBAI), under the framework of MLM. The emBAI algorithm first assumed the marker effect as the random effect and adopted the model transformation of FASTmrEMMA to whiten the covariance matrix of the polygenic and environmental noise, then employed emBayesA to estimate the GEBV. The results of this study show that population structure and polygenic background control are crucial for emBAI in GS, particularly for the accuracy of estimation and AUC of coefficients under different levels of genetic background. It demonstrates that controlling population structure and kinship indeed improves the ability of GEBV estimation and provides theoretical support for GS. Similarly, this idea of background control was applied to emBC, emRR, emEN and emML. The results showed that all the new methods retain computing efficiency and improve the estimation and prediction from the perspective of correlation, MSE and MAE.

In this study, the proposed new method emBAI under the Bayesian framework has the comment merit of a faster computing speed than MCMC-based Bayesian methods. To compare the computing efficiency, all the simulated datasets were reanalyzed by the MCMC-based method BayesA. The results intuitively indicated the computational advantage of the emBAI method. BayesA had a more obvious computational burden as the sample size increased. Taking the second simulation as an example, emBAI took about 2 s for the estimation of 10,000 SNP effects and 500 individuals, and 5 and 10 s for 1000 and 2000 individuals on average, whereas MCMC-based BayesA took more than 20, 40 and 70 s in each dataset of 500, 1000 and 2000 sample sizes, respectively. Moreover, the number of iterations in emBAI was 200, and the iterations ranged from 30 to 120, satisfying the convergence criterion. Compared with the other established EM Bayesian methods, the computing time of emBAI presented an approximately linear increasing trend, while emEN showed an exponential increase. This means that emBAI is more stable and flexible in high-dimensional datasets analysis. In breeding practice, multi-trait GS models have been proposed by utilizing the correlation information among multiple traits, and the advantages of increasing the predictive ability and efficiency are shown in multi-trait with low heritability. The statistical methods of multi-trait GS models will be central to this further work.

## Figures and Tables

**Table 1:** The confirmed genes (top 20) using five methods, including emBAI, emBA, emRR, emML and emBC for LD and FT22 in *Arabidopsis* natural population.

Trait	Chr.	Position	Gene	Method
LD	1	3760729	AT1G11190, BFN1, ENDO1	emBA
	1	3765344	AT1G11190, BFN1, ENDO1	emBAI, emML
	2	13869948	AT2G32700, LUH, MUM1	emRR
	2	16020457	AT2G38185, APD1	emBAI
			AT2G38195, APD4	emBAI
			AT2G38220, APD3	emBAI
	4	14001595	AT4G28190, ULT, ULT1	emBC, emRR
	5	18614010	AT5G45890, ATSAG12, SAG12	emBC
FT22	1	9072307	AT1G26260, CIB5	emML
	2	16020457	AT2G38185, APD1	emBAI, emBC
			AT2G38195, APD4	emBAI, emBC
			AT2G38220, APD3	emBAI, emBC
	2	13869948	AT2G32700, LUH, MUM1	emRR
	4	17263477	AT4G36620, GATA19, HANL2	emBC, emRR
	5	22328009	AT5G55020, ATMYB120, MYB120	emBAI
	5	6844104	AT5G20240, PI, PISTILLATA	emBAI
	5		AT5G20280, ATSPS1F, SPS1F, SPSA1	emBAI
	5	13923880 13923160 13931089 13930569 13927017	AT5G35750, AHK2, HK2	emBA
	5	17219669	AT5G42900, COR27	emBA
	5	9808482	AT5G27650, PDP1	emBA
	5	17463517 17478636	AT5G43510, ATLURE1.2, CRP810_1.2, LURE 1.2	emBA

## Data Availability

Data recorded in the current study are available at http://www.arabidopsis.usc.edu/ (accessed on 22 January 2022).

## Abbreviations

GS, genomic selection; SNP, single nucleotide polymorphism; QTN, quantitative trait nucleotide; GEBV, genomic estimation of breeding value; MSE, mean square error; MAE, mean absolute error; AUC, the area under the receiver operating characteristic curve; TAIR, The Arabidopsis Information Resource; BLUP, best linear unbiased prediction; GBLUP, genomic BLUP; LASSO, least absolute shrinkage selection operator; EM, expectation maximization; MCMC, Markov chain Monte Carlo; MLM, mixed linear model; emBA, expectation maximization BayesA; emBC, expectation maximization BayesC; emRR, expectation maximization Bayesian ridge regression; emML, expectation maximization maximum likelihood; emEN, expectation maximization elastic-net; MAF, the minor allele frequency.
